# Analysis of the Possibility of Using Slags from the Thermal Treatment of Municipal Waste as Potential Component of Cement—Case Study

**DOI:** 10.3390/ma14216491

**Published:** 2021-10-29

**Authors:** Monika Czop, Beata Łaźniewska-Piekarczyk, Małgorzata Kajda-Szcześniak

**Affiliations:** 1 Department of Technologies and Installations for Waste Management, Faculty of Energy and Environmental Engineering, The Silesian University of Technology, Konarskiego 18, 44-100 Gliwice, Poland; mkajda@polsl.pl; 2 Department of Building Processes and Building Physics, Faculty of Civil Engineering, The Silesian University of Technology, Akademicka 5, 44-100 Gliwice, Poland; beata.lazniewska@polsl.pl

**Keywords:** waste, slag, reuse, recycling, mortar, expansion (swell)

## Abstract

In Europe there are nearly 500 incinerators. There are over 2000 of them in the world. It is estimated that the combustion of 1 ton (Mg) of waste produces about 250–300 kg of slag. Due to the large amounts of this waste, the construction industry’s demand for raw materials and the reduction of CO_2_ emissions, research was undertaken to use slags as a cement component. The problem was complex because slags generated in the thermal treatment of municipal waste have different chemical compositions and physical properties and contain variable amounts of impurities. The choice of chemical analyses of slag was dictated by the potential influence on the properties of cement mortars. The total moisture of raw slag (4–10%), the bulk density (600–1267 kg/m^3^) and the specific surface after grinding (over 3000 cm^2^/g) were determined. The pH (11.9) and the content of sulphates (3.5% by weight), chlorides (0.3% by weight) and selected heavy metals (Cd, Cu, Fe, Mn, Zn, Pb) were measured in the aqueous extract. The obtained results of the washing test were compared with the values resulting from the currently binding legal regulations. In the next step, cement mortars with 30% addition of tested slags were designed and made. The article presents the results of compressive strength tests, which were compared with the results of samples without the addition of slag. The addition of slag to the cement mortar decreased S_MSWI 1 by 64% and S_MSWI 2 by 31%. The high loss of strength and the swelling of the S_MSWI 1 test led to the activation of the NaOH slag. In the endurance test, an increase from 16 to 32 MPa was recorded. Preliminary studies show that the addition of slag in the cement mortar allows obtaining the strength at the level of 30–32 MPa.

## 1. Introduction

The generation of municipal solid waste (MSW) is inextricably linked with the everyday existence of a human being. Every year, an increase in the stream of MSW is observed. In 2019, 225 million Mg of MSW was generated in the EU–27 [1], corresponding to 512 kg per capita, increasing 3.3% compared to 2018. The amount of MSW per capita in individual countries varies significantly, from 280 kg per capita inhabitant in Romania to 844 kg per inhabitant in Denmark [1]. On the other hand, in Poland, 336 kg was produced per capita in 2019. While Poland is in the group of countries with the lowest rates, it does not change that we are becoming a more and more consumption and waste based society [2].

In Poland, in 2019, 3.19 million Mg of generated MSW (25%) was directed to recycling (material and organic). Forty-three percent of generated MSW (5.48 million Mg) was landfilled. On the other hand, thermal transformation with energy recovery was subjected to 22% (2.72 million Mg), and 0.20 million Mg (approximately 1% of generated municipal waste) was sent for disposal by a thermal transformation without energy recovery. It should be added that since 2013 there has been an upward trend in the thermal treatment of waste with energy recovery [2].

Due to the growing amount of municipal waste generated, several executive acts have been introduced, which deal with, among other things, the level of recycling or recovery of, e.g., energy. An important document seems to be the European Commission (EC) Communication of 2017 on energy recovery from waste in the Circular Economy (CE is a concept aimed at the rational use of resources and limiting the negative environmental impact of manufactured products [3]). The current waste management system, by Directive (EU) 2018/851 of the European Parliament and of the Council of May 30 2018 [4] provides for energy recovery, which, following the waste management hierarchy, covers only those wastes that have lost their properties, qualifying them for recycling and reuse. One of the elements of the waste management system is Municipal Waste Thermal Treatment Installations (MSWIs).

The act on maintaining cleanliness and order in municipalities (Journal of Laws of 2021, item 888) [5] abolished the 30% limit on thermal treatment wasted from municipal waste processing. The introduced change freed the market of waste incineration plants, which had been limited so far. Local governments see the introduced amendment as the possibility of managing the combustible fraction of municipal waste in local heating systems. Currently, we have a big problem with the optimal management of the municipal waste stream because it contains a lot of high-calorific fractions. The introduced changes may contribute to comprehensive waste management.

On the one hand, the amendment will contribute to the optimal management of the combustible fraction. Still, on the other hand, it will contribute to the formation of significant amounts of secondary waste, including slags, which should be reused to achieve the objectives of the circular economy (circular economy). The idea of the circular economy does not support thermal degradation because it is a loss of material but says that waste for one sector may be a raw material for the other sector [3]. Such an approach is close to the discussed case. For example, waste for MSWI can be a valuable raw material for cement plants. However, this requires research and cooperation between MSWI and the cement industry.

While incineration, in line with the current waste hierarchy [4], is the final disposal element and should not be treated as an alternative to recycling, it should be the worst alternative to storage. Incineration of waste brings several advantages, including reducing the mass and volume of waste and producing heat or electricity. As a result of combustion processes, secondary waste is generated, which in recent years has become the subject of many studies in the context of economic use [6,7]. About 0.25 Mg of slag per 1 ton of incinerated waste is produced and about 0.075 Mg per 1 ton of incinerated fly ash waste, dust from dust removal, filter cakes and gypsum from flue gas cleaning processes [6,7].

Recently, the interest in the use of waste from thermal processes in construction has increased significantly. This direction of using post-process waste is consistent with the principles of sustainable development and the idea of a Circular Economy (CE) implemented in Europe. After appropriate preparation, wastes generated in the combustion process (i.e., slag, fly ash and bottom ash) meet the requirements set by the construction industry. Additionally, they enable economic and environmental benefits to be achieved. Using this type of waste (e.g., slags) reduces the consumption of ordinary cement, which significantly affects the consumption of non-renewable, natural raw materials needed for its production. It also reduces anthropogenic CO_2_ emissions to the atmosphere and reduces the energy consumption needed for its production. The factors mentioned above improve the condition of the environment in Poland, which ultimately translates into global effects.

Additionally, studies [8] confirm the possibility of using slag from the incineration of MSW as an artificial aggregate. According to publication [8], the dominant direction in using slags in Poland in 2016 is mostly storage. To a lesser extent, they are used as an additive for the production of road aggregates.

Slags resulting from combustion can be used in construction, saving natural resources. However, it is worth remarking that the building materials produced based on such slags are most often characterised by low strength and therefore are mainly used in local construction roads. Regardless of their use, the resulting final product must not be harmful to the environment. Based on the literature, it can be concluded that the content of chlorides and heavy metals in slags from municipal garbage incinerators is relatively not great. In addition, it is assumed that any undesirable substances will be somewhat sunk in the structure of concrete.

Nevertheless, any even minor risk of leakage of harmful substances should be assessed at the production stage of the materials produced and during their operation and subsequent degradation. When preparing concretes, based on aggregate sew from the incinerator, the relationship between cement and the aluminium and zinc contained in the waste is particularly undesirable. It is known that these reactions cause the phenomenon of swelling and even cracking of concrete. To counteract these phenomena, slags from the incinerator, before being used in the production of building materials, should most often be seasoned and processed. Waste treatment can take various forms depending on the initial composition and future importance (seasoning, rinsing, washing, sodium hydroxide action, heavy metal removal, glazing). Many studies emphasise the need to use such treatments, or at least to pre-wash the obtained slag. Numerous studies show that only the processed (cleaned) combustion product has the appropriate chemical and physical properties ensuring the expected quality of concrete. However, while environmentally friendly, the materials often obtained by the authors were characterised by low strength. One of the most common problems was reducing the aluminium and the glass contained in the waste after incineration. The furnace slag from current production contained particles of unburned coal and sometimes significant amounts of sulphur oxides. Therefore, it should not be applied to the concrete. Burnt furnace slag aggregate should be applied to concretes provided that the sulphur content does not exceed 3%. The search for an environmentally friendly concrete mix with good mechanical properties is still valid. Because the waste treatment represents a highly actual topic, several researchers have studied the possibility of recycling MSWIA materials in concrete and cement manufacturing, both as aggregates or mineral additions [9,10,11,12,13,14,15,16,17]. MSWI bottom ash and fly ash have also been used as raw materials for manufacturing cement, ceramics, bricks or tiles.

Publications [18,19] analysed the possibilities of using MSWI ashes as a component of cement. Paper [20] describes the results of research aimed at studying the effect of replacing part of Portland cement with fly ash and bottom ash, both from municipal solid waste incinerators (MSWIs). The studied MSWI ashes exhibit a high concentration of chlorides and sulfates, which is an unfavourable feature for a potential concrete additive [21,22]. Publication [23] indicated that the MSWI slag structure is loose and irregular, and its main component is SiO_2_. The SiO_2_ and Al_2_O_3_ in fly ash and slag participate in the hydration reaction of cement and can increase concrete strength. It is thus confirmed that fly ash and slag generated by waste incineration can replace cement and coarse aggregate in appropriate proportions. This is an effective method to solve the problem of scarcity of solid waste landfill space.

However, there are still few studies in the literature on the effect of ground slag as a component of cement. The reactions between cement, aluminium and zinc in the waste are particularly undesirable [24,25,26,27]. The reasons and course of these reactions have been described in detail in the literature. It is known that these reactions cause concrete swelling and even cracking. To counteract these phenomena, slags and ashes from incineration plants are mostly seasoned and processed before using them for the production of building materials. Depending on the initial composition and future destination, waste treatment may vary forms (seasoning, rinsing, washing, treatment with sodium hydroxide, removal of heavy metals, glazing). Many studies emphasise the need to use such treatments, or at least preliminary rinsing of the obtained slag. Numerous studies show that only the processed (cleaned) combustion product has the appropriate chemical and physical properties, ensuring the expected quality of concrete. However, while environmentally friendly, the materials often received by authors were characterised by low-level endurance. One of the most common problems was the need to reduce the aluminium and the amount of glass contained in the waste after incineration and limit the release into the environment of potentially harmful substances. The authors concluded that improperly selected, unseasoned slag may cause concrete swelling based on the conducted research. Therefore, the authors propose to use alkaline activation of slag to eliminate the swelling of the mortar with its participation. The results of the trials are analysed in this article. Similar research of this scope has not been conducted so far. 

## 2. Materials and Methods

### 2.1. The Analysed Installation of Thermal Processing of Municipal Waste

Thermal treatment installations for municipal waste have been a common element of the waste management system in European Union countries for many years. It is estimated that in Europe there are approximately four hundred installations for thermal degradation of waste. Thermal degradation of MSW in Europe is carried out in combustion chambers equipped with various grates. The dominant solution in installations for thermal degradation of municipal waste in Europe is grate incineration. All technologies are based on installations that perfectly clean exhaust gases from all kinds of air pollutants. Solid waste, slags, ash and solid waste are subjected to flue gas treatment [28,29,30].

The article presents the two largest installations of MSWI operating in Poland, which started in 2016 [31,32]. On the one hand, the installation choice was dictated by the similarity to popular European installations—grate combustion. On the other hand, there are differences in the way of collecting post-process waste. The method of collecting post-process waste, including case studies, translates into their physicochemical properties. It should be mentioned here that the installations in question operate in agglomerations where the approach to waste management is holistic. Compliant with the applicable hierarchy of waste management, where recycling is a priority, energy recovery is an alternative to landfilling.

**Figure 1:**
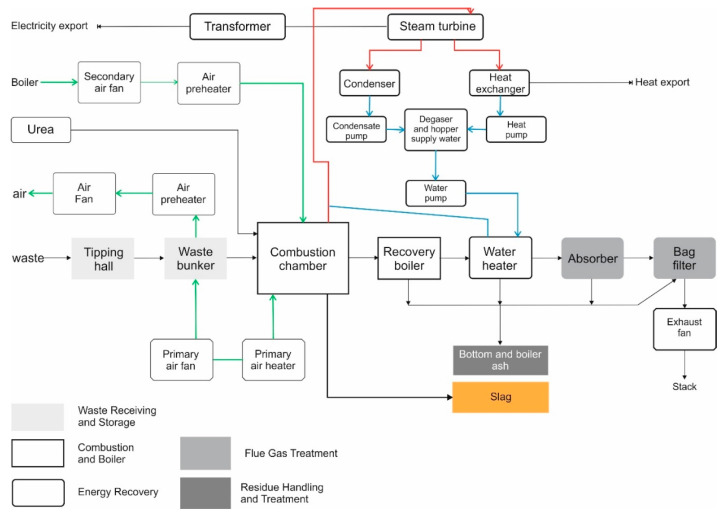
Simplified scheme of an analysed MSWI 1 incinerator.

Mixed municipal waste and other waste resulting from mechanical processing of municipal waste (after waste recovery processes, i.e., material, large-size, post-repair waste) are sent to the Municipal Waste Thermal Conversion Installation 1 (MSWI 1) [31]. The annual capacity of the MSWI 1 installation is 220,000 Mg, with the calorific value of waste equal to 8.8 MJ/kg. The Installation is equipped with two technological lines with a capacity of 14.1 Mg/h [31]. The thermal power of the Installation is 35 MWt, and the electric power is 10.7 MWe. Post-process waste is generated in the combustion process: Slag and bottom ashes, boiler dust and fly ashes, and solid residues from flue gas cleaning. The residues from the waste incineration process constitute about 25% of the input stream to MSWI 1. Slag and bottom ash are managed through their valorisation [31]. The first stage of valorisation is preliminary seasoning carried out in a closed building. The goal is to dehydrate and stabilise. After this stage (approximately two weeks), the slag is treated, during which fractions of appropriate size, as well as ferrous and non-ferrous metals, are separated. Finally, the obtained fractions are transferred to the slag seasoning warehouses. Figure 1 presents the block technological diagram of MSWI 1 [31].

**Figure 2:**
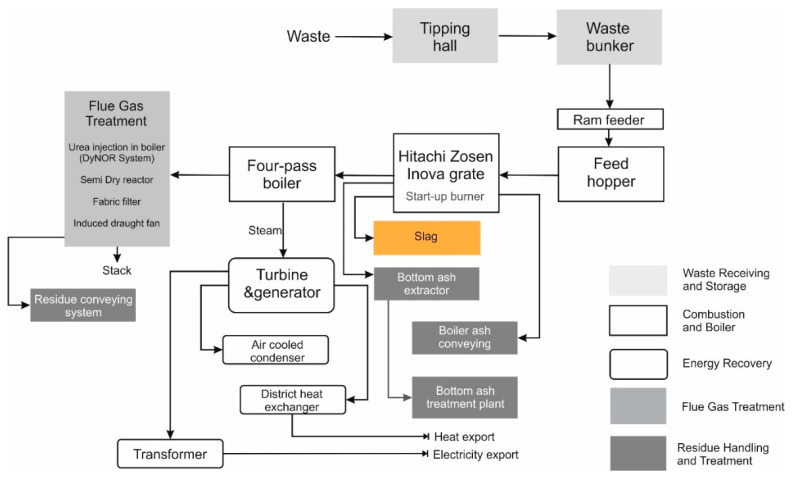
Simplified scheme of an analysed MSWI 2 incinerator.

The Municipal Waste Incineration Plant 2 (MSWI 2) is adapted to convert non-hazardous mixed municipal waste. The annual capacity of the MSWI 2 installation is 210,000 Mg, with the calorific value of waste being 8.4 MJ/kg [32]. The Installation is equipped with two combustion lines with a capacity of 13.5 Mg/h. The thermal power of the Installation is 34 MWt, and the electric power is 15 MWe. The waste incineration process produces post-process residues. However, they are safely managed on the site of the Installation and undergo recovery processes. At the installation site, ferrous and non-ferrous metals are recovered from the slag [32]. The remaining fraction is seasoned. Figure 2 shows the block technological scheme of MSWI 2.

### 2.2. Materials

The materials used for the tests were slags. Slag is a by-product of mixed municipal waste combustion in grate furnaces; it consists of non-flammable substances (water-insoluble silicates, aluminium and iron oxides). The analysed slags came from two MSW incineration plants with the highest processing capacity, according to the Catalog of Wastes [33,34], with the code 19 01 12 being bottom ash and slag other than those mentioned in 19 01 11.

**Figure 3:**
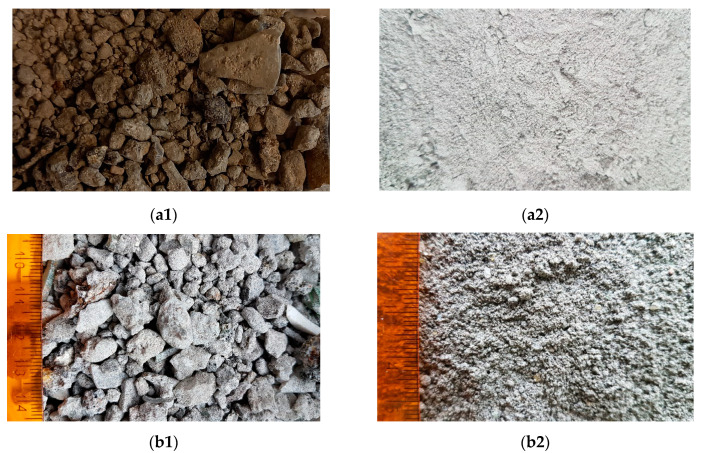
Analysed slags: Slag MSWI 1: (**a1**) raw, (**a2**) ground; Slag MSWI 2: (**b1**) raw, (**b2**) ground.

In both considered cases, the slags are managed through valorisation in the Slag Valorization Junction. The first stage of the valorisation process is initial seasoning to dehydrate and stabilise the slag. Then, appropriate size fractions and ferrous and non-ferrous metals are separated using a magnetic and induction separator [22,23]. Figure 3 presents the tested slags in the raw state (a1,b1) and after mechanical treatment (a2,b2).

### 2.3. Analytical Methods

#### 2.3.1. Methodology of Consumption Preparation and Analysis of Its Properties

The environmental aspect of the slag test procedure included:

- Testing the physical and chemical properties of the analysed slags from the process of thermal degradation of municipal waste;
- Preparation of a water extract from slags and assessment of the degree of an environmental nuisance.

In the first analytical step, the water content in the slags submitted for testing was determined based on the standard 15934: 2013-02 [35] and the bulk density following the PN-EN 1097-6: 2013-11 standard [36].

The tested ash samples were subjected to initial grinding in a ceramic ball mill to obtain the material with a high degree of homogenisation. The ash samples prepared in this way were subjected to selected physicochemical analyses. The homogenised samples were subjected to selected physicochemical analyses. The specific surface area was determined [37], as were the losses on ignition of dry matter [38].

The samples were also tested for the content of the following elements: Total carbon (C)—high-temperature method [39]; organic carbon (TOC)—the method involves the oxidation of an organic substance with the use of potassium dichromate as an indicator [40]; total nitrogen (N)—the Kjeldahl method consisting in converting nitrogen into amine compounds [39]; total sulfur (S)—using the Eschka method [41]; and chlorine (Cl)—the Mohr method with the use of Eschki mixture [42]. Furthermore, the flame emission spectrometry method determined sodium, calcium, potassium, lithium and barium [43]. In addition, Perkin Elmer inductively excited plasma mass spectrometer (ICP MS) was used, which allows the determination of elements excited in argon plasma to check the content of heavy metals of the dry mass of the sample [44].

The performance of water extracts from the slag was performed according to PN-EN 12457-2: 2006 standard [45]. First, a representative laboratory sample was prepared from the collected waste weighing 2 kg. For analysis, the tested waste was sieved through sieves with a mesh size of 2 mm; from the sample prepared in this way, a water extract was prepared with the ratio of liquid to solid phase L/S = 10 dm^3^/kg (basic test). The leaching liquid was distilled water with a pH of 7.1 [46] and specific conductivity of 61.18 µS/cm [47]. The extract was then shaken on a laboratory shaker for 24 h, and the suspension was filtered. The analysis of the water extracts included several determinations:

- Organic carbon content was determined using the Elementar Vario TOC Cube analyser;
- A pH of the solutions and the electrolytic conductivity was determined using an El-metron CPC-501 apparatus [46,47];
- Chloride contents were determined by the Mohr method with the use of silver nitrate (V) as a titration reagent and potassium chromate (VI) as an indicator [48];
- Sulphates (VI) (SO_4_^2−^) was carried out using the gravimetric method with barium chloride [49];
- Sodium, calcium, potassium, lithium and barium content in water extracts were determined by flame emission spectrometry [43];
- Phosphorus content was investigated by the spectrophotometric method based on the absorbance of the complex at a wavelength of 690 nm [50].

The Perkin Elmer inductively excited plasma mass spectrometer (ICP MS) was used, which allows the determination of elements excited in argon plasma [44] to assess the content of heavy metals in the aqueous extract.

#### 2.3.2. Composition, Methodology for Preparing Mortars and Evaluating Their Properties

In the research, concrete mortars with a binder composition are:

- Portland cement CEM I 42.5R (from now on referred to as CEM I);
- Portland cement-based binder CEM I 42.5R with 30% added ground slag from MSWI 1 incineration plant (from now on referred to as CEM I + 30% S_MSWI 1);
- Portland cement-based binder CEM I 42.5R with 30% added ground slag from the MSWI 2 incinerator (from now on referred to as CEM I + 30% S_MSWI 2);
- Portland cement-based binder CEM I 42.5R with 30% added ground slag from the MSWI 1 incinerator (CEM I + 30% S_MSWI 1 after alkaline activation).

Concrete mortars were made following the methodology described in EN 197-1:2012 [51]. The composition of the tested mortars is presented in Table 1. Characteristics of cement are presented in Table 2. Te used sand and according to EN 196 has a standardised amount and size of grains. The characteristic of CEN Standard Sand is its specific grain size distribution. It ranges between 0.08 and 2.00 mm. The grain size distribution is drawn in the picture (cumulated sieve residue in % vs. squared mesh size in mm) and listed below. The maximum moisture content is 0.2%. The sand is portioned in bags of 1350 (±5) g. According to EN 196-1, mortar prisms for compressive strength testing are produced with a mixture of 450 (±2) g cement, 225 (±1) g water and one bag of 1350 (±5) g CEN Standard Sand.

The alkaline activation of the slags consisted in subjecting them to a five-molar NaOH solution for 48 h. Subsequently, the slag was rinsed vigorously and dried before the grinding process.

The consistency of the mortars was checked according to the table method described in EN 1015-3 [52]. Then, the volume change of cement with slag was conducted using a standard slurry according to EN 196-3 [53]. Finally, after 28 days of carrying in water, the mechanical properties of mortars were investigated according to standard EN 196-1 [54]. 

## 3. Results and Discussion

### 3.1. Physical and Chemical Properties of MSWI Slag

Table 3 presents the basic technical properties of the tested MSWI slags. The absolute humidity of slag MSWI 1 was high, equal to 10%, while the moisture content in the sample slag MSWI 2 was of the order of 4.5%; these values are much higher than recommended in the standard (≤1.0%) [19]. Elevated humidity may influence pozzolanic activity. Exceeded humidity may contribute to the cracking of the concrete swelling process. The moisture content of the slags can be lowered by extending the seasoning time at the manufacturer premises. Extending the seasoning process is possible based on the consultations carried out in the analysed Ministry of Interior and Administration. 

Bulk density slag MSWI 1 was 600 kg/m^3^, while for slag MSWI 2 it was in the order of 1700 kg/m^3^, a value slightly higher than the value of the commonly used Portland cement (900–1500 kg/m^3^).

The undesirable components of a potential mineral additive include too high a content of sulfur compounds, chlorine and unburned carbon. The high content of unburned carbon (C > 5%) may increase the water demand and reduce the frost resistance of mortars or concrete with its participation. In the analysed slags, the content of unburned carbon was below 5% [55]. The content of chlorine, sulfur and organic carbon in the tested slag was below 1%. Low contents of the elements mentioned above (S, TOC, Cl) translated into their trace leachability.

The conducted research on the content of heavy metals in slags confirmed the relatively high level of most trace elements (Table 3). The content of heavy metals in the tested slags generated in the municipal waste incineration process had the following sequences:

- Slag MSWI 1 (Cu > Zn > Pb > Cr > Ni > V > Cd > AsTl > Hg),
- Slag MSWI 2 (Cu > Zn > Pb > Cr > Ni > V > As > Cd > Tl > Hg).

Among the analysed metals, the highest content for all tested samples was recorded for Cu in the range of 1918.0–21,608.0 mg/kg and Zn in the range of 1621.0–2797.0 mg/kg, and the lowest value was recorded for mercury 0.04–0.24 mg/kg.

**Figure 4:**
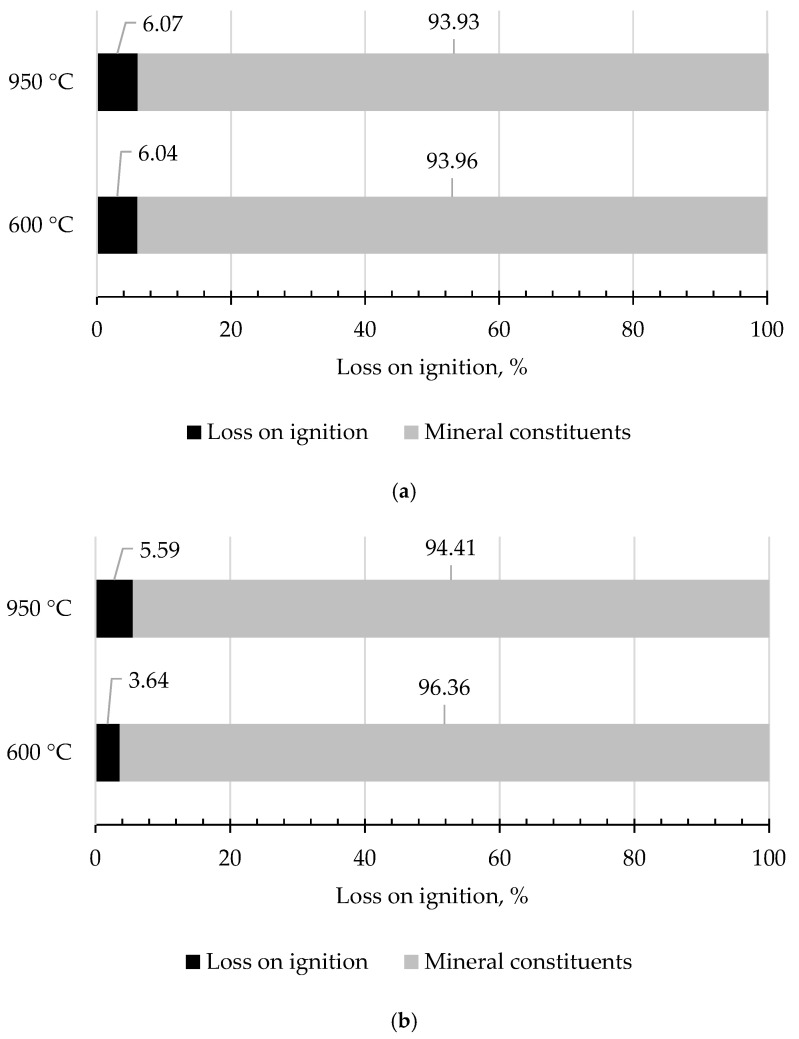
Loss on ignition (LOI), (**a**) slag MSWI 1, (**b**) slag MSWI 2.

Figure 4 shows the results of the loss on ignition (LOI) for the tested slags. LOI was determined by heating the slag samples to constant weight in a muffle furnace at two temperatures: 600℃ and 950℃ in an oxidising atmosphere. In the considered cases, it was found that LOI in 600 °C meets the criterion of admitting to a landfill for non-hazardous and inert waste (LOI ≤ 8%) [20,55]. The ignition losses of tested slags were determined by roasting the samples at the temperature of 950 °C for one hour. This parameter is essential due to its application in construction. The acceptable LOI limit for GBFS is ≤3% [19]. High losses on ignition in furnace slags may result in deterioration of the workability of the concrete mix. LOI values for MSWI 1 and MSWI 2 slags amounted to 6.07% and 5.59%, respectively.

In the following research step, the chemical analysis of the tested furnace slags was carried out. The oxide composition and heavy metal content of MSWI slags were determined; the results obtained are presented in Table 3 and Table 4. The tested furnace slags (slag MSWI 1 and slag MSWI 2) are characterised by a similar oxide composition. The primary phase component of MSWI furnace slags is silicon SiO_2_. A high silica content (SiO_2_ > 50%) may translate into a correspondingly high pozzolanic activity. The CaO content meets the requirements for ground granulated blast furnace slag (CaO ≤ 18%). Table 4 shows the chemical composition of the tested furnace slags and the ratio of individual oxides according to the standard characterising granular blast furnace slag (GBFS) [46].

According to the requirements of the standard [46], slag for construction should consist of at least two-thirds of the mass of the sum of calcium oxide (CaO), magnesium oxide (MgO) and silicon oxide (SiO_2_). Unfortunately, the composition of the bottom slag does not reach the required level in both cases. Table 5 presents the properties of granulated blast furnace slag about the requirements of PN–EN 197–1 “Cement-Part 1. Composition, requirements and compliance criteria for ordinary cement” [51].

The weight ratio (CaO + MgO)/SiO_2_ should be above 1.0%. In both tested cases, the slag shows a value lower than the required value. The activity coefficients calculated based on the chemical composition are low and amount to 0.36% for MSWI 1 slag and 0.24% for MSWI 2 slag. They bind and harden due to reactions and hydration processes and remain durable, even underwater.

### 3.2. Assessment of the Level of Leaching of Pollutants from the Tested Slags to the Natural Environment

MSWI slag, considered an alternative material in construction, cannot threaten the environment and people. Environmental studies have been carried out to ensure the safe use of this material. Table 6 show the leachability of selected impurities from the tested furnace slags, which may be an environmental nuisance and adversely affect the mechanical properties of the concrete mix, which may translate into concrete durability. Post-process waste from municipal waste incineration may pose an environmental problem due to its high salt content, mainly chloride salts and sulphates. The leachability of chlorides from the tested furnace slags does not exceed the permissible level for storage at landfills for inert waste in the case of the slag sample MSWI 2. In the case of the slag sample MSWI 1, the chloride content does not exceed the permissible level for storage in non-hazardous and inert waste landfills. The level of sulphate leaching for the MSWI 1 slag does not exceed the permissible value for storage at landfills for inert waste.

In contrast, for the MSWI 2 slag, the exceedance of the acceptable standard for inert waste is insignificant. Furthermore, when analysing the obtained results in terms of chemical requirements, it should be noted that the content of chloride ions in the tested furnace slags does not exceed 0.5%, and the content of sulphate ions does not exceed 1.5%. Therefore, it can be concluded that the tested furnace slags will not constitute a significant environmental nuisance at the time of their reuse.

The levels of leachability of heavy metals Cd, Co, Cu, Cr, Hg, Ni, Pb, Mn and Zn were determined by measuring the concentrations of individual elements (ASA method) in water extracts of these materials, prepared with a solid/water ratio of 1/10 [20]. The leachability of heavy metals in the tested slags was very low. Only the Pb content for the sample Slag MSWI 2 exceeded the limit value for waste stored in inert landfills by 0.1 mg/kg. The content of the remaining metals (Zn, Cd, Ni) did not exceed the permissible value. Moreover, the amount of Ba, Cu, Cr, Co, Fe and Mn was below the quantification threshold for the slag sample MSWI 1. Therefore, the content of all determined heavy metals was below the quantification threshold.

### 3.3. Assessment of the Possibility of Applying MSWI Slag as a Cement Component

At present, no standards specify the requirements for MSWI slag as an additive to cement. Therefore, for this article, the requirements for ground granulated blast furnace slag (GBFS), used as a type II additive in the concrete composition, were used as a reference point for considerations [57]. Standard requirements for GBFS are presented in Table 7.

The size of the specific surface has been defined. The specific surface area should be ≥2750.0 cm^2^/g. The specific surface area of both tested furnace slags after grinding in a ball mill meets the GBFS standard. The considered slags also meet the chemical requirements about the content of MgO, SO_3_, Cl^-^ for Slag MSWI 2. However, exceedances are visible for LOI. The permissible LOI limit for GBFS is ≤3% [19]. High losses on ignition in furnace slags may result in deterioration of the workability of the concrete mix. In slag MSWI 1, LOI was at the level of 6.07%, and in slag MSWI 2, the value was at the level of 5.59%. The moisture content of both slags is also exceeded. This parameter can be lowered by extending the seasoning period.

### 3.4. Analysis of Fresh and Hardened Mortars Properties

Test results in Table 8 indicated that the analysed cement paste with 30% volume of MSWI 1 and MSWI 2 slags does not exceed 10%, following the requirements of standard EN 197-1:2012 [42]. Moreover, the research results proved that alkaline activation of slag MSWI 1 reduces changes in the cement paste volume. Therefore, instead of using a relatively expensive and environmentally burdensome alkaline activation, it is enough to subject it to a sufficiently long-term seasoning.

The research results proved that alkaline activation does not affect the consistency of the mortars. Interestingly, the slag was not activated with alkali but had different chemical characteristics and did not cause swelling.

**Figure 5:**
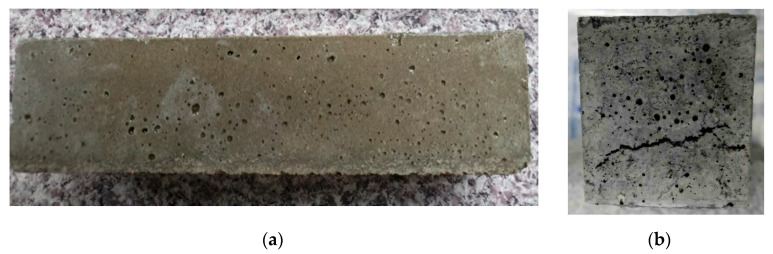
Portland cement-based binder CEM I 42.5R with 30% added ground slag from MSWI 1 incineration plant (**a**) front, (**b**) side.

**Figure 6:**
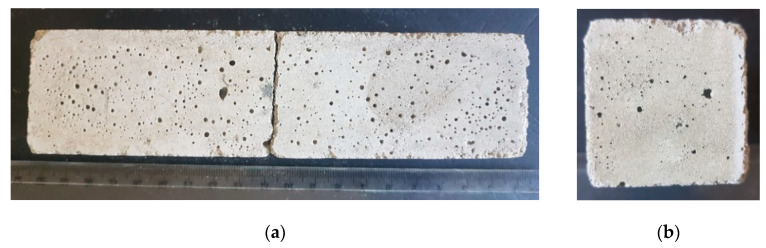
Portland cement-based binder CEM I 42.5R with 30% added ground slag from MSWI 2 incineration plant (**a**) front, (**b**) side.

**Figure 7:**
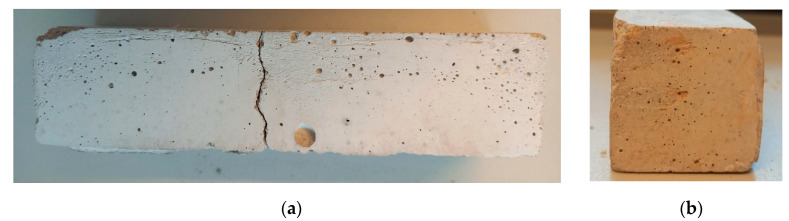
Portland cement-based binder CEM I 42.5R with 30% added ground slag from MSWI 1 incineration plant—after alkaline activation in 5M NaOH (**a**) front, (**b**) side.

The comparison of the mechanical properties of mortars indicated that an increase in the volume of the mortar causes the deterioration of the mechanical strength (Table 9). Furthermore, in the sample of the inverted pipe, numerous cracks in its size were also found (Figure 5). Nevertheless, no cracks were found in the sample with the consumption subjected to alkaline activation and in the sample with MSWI 1 (Figure 6 and Figure 7). Therefore, high-energy grinding is an effective way to achieve high speedway finesse rates. High-energy grinding affects the use of MSWI slag as an additive without the pozzolanic effect, but only when inert, in the amount used in the case of CEM II and amounting to about 30%, so as not to reduce the strength of cement mortars too much. In study [20,58], pozzolanic slag was used, and it was shown that concerning the improvement of both short-term and long-term compression the strength of the slag cement grout was achieved at 45% replacement of CEM I cement high-energy ground slag.

The unfavourable effect of MSWI can be explained by chemical reactions to non-ferrous metals, especially aluminium and zinc, which increase the volume of mortar and concrete containing alkaline compounds [12,18,50]. The most incredible nuisance in the possible use of slag is the constant need to check and reduce the amount of aluminium, glass and unburned carbon, as well as chlorides, zinc and sulfur. Under alkaline conditions, which occur during cement hydration, metallic aluminium can form hydroxides or lead to the emission of hydrogen. The research results given in Table 6 indicate that zinc in MSWI 2 is higher than in MSWI 1. Moreover, the total carbon amount is higher in MSWI 2 than in the case of MSWI 1 (Table 3), which is also connected with the change in volume of mortar. When the aluminium volume is high in MSWI, the purpose of preventing volume changes, and more precisely swelling, of concretes containing MSWI materials, is their initial activation in sodium hydroxide until the complete release of hydrogen from their volume, according to the reaction:

NaOH + Al + H_2_O→NaAlO_2_ + 3n/2 H_2_ (1)

Pera et al. [12] applied this method before using MSWI waste in concrete. The MSWI waste was immersed in sodium hydroxide solution for 15 days, and then it was washed and dried. Concrete containing waste prepared in this way did not change its volume.

A large amount of sulfur in the slag can also contribute to the swelling of cement mortars due to reactions with lime. Therefore, low-alkali cement is recommended as a complement to MSWI slag mortars. The research results presented in Table 9 confirm the effectiveness of replacing cement with a reduced amount of alkali to reduce the swelling of mortars and concretes. However, a critical review [59] of alkaline-activated slag performance suggests that although alkalic activators give high concrete strength, their detrimental effects limit its large-scale use. Therefore, replacing the NaOH activator with sodium carbonate is proposed because it gives similar efficiency with activated sodium hydroxide and sodium silicate. In addition, it provides excellent properties of a fresh mix of brick and concrete. As a result, it can be used on a much broader scale due to its ecological nature concerning energy and coal production. Further research ought to take place, especially on the microstructure of mortars, concretes and geopolymers with alkali-activated slag, also with MSWI slag.

## 4. Conclusions

Slag from thermal treatment of municipal waste is not an accessible raw material to use. Still, after appropriate treatment, it can be a good and valuable alternative to non-renewable natural resources. To use MSWI furnace slags in the construction industry, e.g., for the production of building materials or roads, it is necessary, together with manufacturers, to develop a procedure for their cleaning, processing and seasoning. Depending on the input stream to the Installation and its future use, their preparation may take various forms, such as seasoning, rinsing, washing or treatment with sodium hydroxide. Furnace slags require systematic analyses, mainly concerning the chemical composition. Then, it will be possible to make the right decision regarding their use, e.g., for concrete production. After preliminary tests, the furnace slag, formed in the combustion process, analysed in the article, can be successfully used as a substitute for some cement (e.g., 30%) in the so-called lean concrete. Additionally, they can be used in the construction of road foundations.

Reactions between cement and aluminium and zinc contained in the slags may be a barrier to the use of furnace slags. An additional difficulty may be caused by the heterogeneity of the furnace slag and a significant content of harmful components (i.e., unburned coal, sulfur compounds). The mentioned components may cause reduced strength of concrete with the addition of furnace slag, consequently translating into a limited scope of application. However, it has been proven that the alkaline activation of the slag reduces the swelling of the mortars. Moreover, low-alkali cement is recommended as a complement to MSWI slag mortars. The obtained results allow environmentally friendly use of furnace slags with the possibility of obtaining tight and durable concretes. This method of use may be the best and cheapest long-term solution in waste management and fill the market gap in this area. The search for mineral additives for concrete, including secondary waste from thermal degradation, is still valid. Currently, we are obliged to look for recyclable raw materials to use waste that can be given another life cycle. Thermal degradation of municipal waste is one of the elements of modern waste management, which aims to abandon landfilling completely.

Additionally, they can support local heating installations. Finally, post-process waste, such as slags, can be a valuable aggregate or cement component. The result presented in the article is an introduction to further research and discussion because the problem is will continue in the future, is global and is interdisciplinary.

## Figures and Tables

**Table 1:** Composition concrete mortars.

Type of Waste	Symbol of Mortar	CEM I, g	Water, g	Sand acc. EN 196-1, g
Reference sample from Portland cement 42.5 R	CEM I 42.5 N	450	225	1350
CEM I 42.5 N + 30% Slag MSWI 1	CEM I 42.5 N + 30% S_MSWI 1	315	135	1350
CEM I 42.5 N + 30% Slag MSWI 2	CEM I 42.5 N + 30% S_MSWI 2	315	135	1350
CEM I 42.5 N + 30% Slag MSWI 1, NaOH	CEM I 42.5 N+ 30% S_MSWI 1 after alkaline activatin in 5M NaOH	315	135	1350
CEM I 42.5 N-SR 3/NA + 30% MSWI 2	CEM I 42.5 N-SR 3/NA+ 30% S_MSWI 2	315	135	1350

**Table 2:** Chemical and physical characteristics of cement.

Physico-Chemical Properties of the Product (Average Values)	CEM I 42.5 N	CEM I 42.5 N-SR 3/NA
Specific surface, cm^2^/g	3548	3132
Start of setting time, min	205	226
End of setting time, min	280	289
Compressive strength after two days, tested following PN-EN 196-1, MPa	20.8	22.9
Compressive strength after 28 days, tested following PN-EN 196-1, MPa	54.5	51.1
Specific density, g/cm^3^	3.17	3.18
The content of SO_3_ sulphates, %	3.0	2.57
Chloride content Cl^−^, %	0.10	0.058
Alkali content (eq Na_2_O), %	0.60	0.51
Al_2_O_3_ content, %	5.0	3.79
C_3_A content, %	5.0	1.29
C_4_AF + 2C_3_A content, %	15.80	18.33

**Table 3:** Basic technical properties of tested slags.

Properties	Symbol	Unit	Slag MSWI 1	Slag MSWI 2
Moisture	M	%	10.00	4.48
Specific surface area	S	cm^2^/g	3065.0	3200.0
Bulk density	ρ_b_	kg/m^3^	600.0	1700.0
Total carbon	C	%	1.44	2.26
Total organic carbon	TOC	%	* blq	0.52
Sulfur	S	%	0.98	0.78
Chlorine	Cl	%	0.39	0.12
Zinc	Zn	mg/kg	2797.00	1621.00
Copper	Cu		21,608.00	1918.00
Lead	Pb		766.00	687.00
Nickel	Ni		73.60	81.00
Chrome	Cr		277.00	342.00
Cadmium	Cd		5.80	3.35
Arsenic	As		5.30	16.50
Vanadium	V		31.80	30.00
Thallium	Tl		<1.00	<1.00
Mercury	Hg		0.04	0.24

* blq–Values below the limit of quantification.

**Table 4:** Content of oxides (%) in the tested materials.

Properties	Symbol	Slag MSWI 1	Slag MSWI 2	GBFS [46]
Silicon dioxide	SiO_2_	50.50	57.90	37.63
Iron(III) oxide	Fe_2_O_3_	5.00	4.97	1.48
Aluminium oxide	Al_2_O_3_	11.30	10.80	6.84
Manganese(II,III) oxide	Mn_3_O_4_	0.11	0.12	-
Titanium dioxide	TiO_2_	0.95	0.50	-
Calcium oxide	CaO	16.50	12.50	45.63
Magnesium oxide	MgO	1.77	1.73	5.33
Sulfur trioxide	SO_3_	1.39	0.74	0.08
Phosphorus pentoxide	P_2_O_5_	1.12	0.74	-
Sodium oxide	Na_2_O	4.16	6.61	0.55
Potassium oxide	K_2_O	0.84	0.95	0.56
Barium oxide	BaO	0.14	0.14	-
Strontium oxide	SrO	0.04	0.06	-

**Table 5:** Properties of tested slags in relation to the standard requirements of PN–EN 197–1.

Requirements of PN–EN 197–1 [42]		Slag MSWI 1	Slag MSWI 2	GBFS [46]
CaO + MgO + SiO_2_	≥67%	68.77	72.13	89.00
(CaO + MgO)/SiO_2_	≥1.0	0.36	0.25	1.18
CaO/SiO_2_	1.3 ÷ 1.4	0.33	0.21	1.03
(CaO + MgO)/(SiO_2_ +Al_2_O_3_)	1.0 ÷ 1.3	0.30	0.21	0.98
(CaO + 1.4·MgO + 0.56·Al_2_O_3_)/SiO_2_	≥1.65	0.50	0.36	1.36
(CaO + MgO + Al_2_O_3_)/SiO_2_	≥1.0	0.58	0.43	1.21

**Table 6:** Leachability of selected contaminants of tested slags, expressed in mg/dm^3^ (with pH exception).

Properties	Symbol	Slag MSWI 1	Slag MSWI 2	The Highest Allowed Value [56]
pH	pH	10.7	7.9	6.0–9.0
Total Organic Carbon	TOC	* blq	* blq	30
Chloride	Cl^−^	360.00	78.00	1000
Sulphate	SO_4_^2−^	47.15	115.74	500
Phosphorus	P	1.19	3.27	2
Potassium	K	59.13	35.46	80
Calcium	Ca	158.53	87.84	** nr
Lithium	Li	0.97	0.27	** nr
Sodium	Na	332.93	110.40	800
The sum of chloride and sulphate	Cl + SO_4_^2−^	407.15	193.74	1500
Bar	Ba	* blq	* blq	2
Zinc	Zn	* blq	0.01	2
Copper	Cu	* blq	* blq	0.5
Lead	Pb	* blq	0.06	0.5
Cadmium	Cd	* blq	0.004	** nr
Chrome	Cr	* blq	* blq	0.1
Cobalt	Co	* blq	* blq	1
Iron	Fe	* blq	* blq	10
Manganese	Mn	* blq	* blq	** nr
Nickel	Ni	* blq	0.02	0.5

* blq—values below the limit of quantification; ** nr—no requirements.

**Table 7:** Requirements to be met by ground granulated blast furnace slag as a cement component.

Parameter	Symbol	Unit	Standard Requirement GBFS [45]	Slag MSWI 1	Slag MSWI 2
Specific surface area	-	cm^2^/g	≥2750.0	3065.00	3200.00
Magnesium oxide	MgO	%	≤18.0	1.77	1.73
Sulfide	S^2−^		≤2.0	-	-
Vitreous phase	-		≥67.0	-	-
Sulfates	SO_3_		≤2.5	1.39	0.74
Loss on ignition	LOI		≤3.0	6.07	5.59
Chloride	Cl^–^		≤0.1	0.36	0.08
Moisture	M_T_		≤1.0	10.00	4.48

**Table 8:** The test results of the change of the volume and consistency of the mortars.

Symbol of Mortar	Change Volume, %	Slump Flow Diameter, mm
CEM I 42.5 N	0.5	170
CEM I 42.5 N + 30% S_MSWI 1	3.0	160
CEM I 42.5 N + 30% S_MSWI 2	0.6	160
CEM I 42.5 N+ 30% S_MSWI 1 after alkaline activation in 5M NaOH	0.6	162
CEM I 42.5 N-SR 3/NA + 30% MSWI 2	1.0	163

**Table 9:** The test results of mechanical properties of the mortars.

Symbol of Mortar	Sample Height, mm	28 Day Compressive Strength, MPa	28 Day Tensile Strength, MPa
CEM I 42.5 N	40	45	6
CEM I 42.5 N + 30% S_MSWI 1	40	16	2
CEM I 42.5 N+ 30% S_MSWI 2	47	31	4
CEM I 42.5 N + 30% S_MSWI 1 after alkaline activatin in 5M NaOH	40	32	4
CEM I 42.5 N-SR 3/NA + 30% MSWI 2	41	30	5

## Data Availability

Not applicable.
